# A Giant Paraovarian Cyst Misdiagnosed as a Distended Bladder: A Case Report

**DOI:** 10.7759/cureus.89271

**Published:** 2025-08-03

**Authors:** Filipa Caires, Catarina Ornelas, Mariana Andrade, Carlos Fernandes, João Horta Antunes

**Affiliations:** 1 Caniço Health Center, Hospital Dr. Nélio Mendonça, Serviço de Saúde da Região Autónoma da Madeira (SESARAM), Funchal, PRT; 2 Santana Health Center, Hospital Dr. Nélio Mendonça, Serviço de Saúde da Região Autónoma da Madeira (SESARAM), Funchal, PRT; 3 Internal Medicine, Hospital Beatriz Ângelo, Loures, PRT

**Keywords:** distended bladder, giant paraovarian cyst, gynecologic mass, paraovarian cyst, pelvic mass

## Abstract

Paraovarian cysts (PCs) are uncommon adnexal lesions that often present with nonspecific or absent symptoms, making clinical diagnosis challenging. When they exceed 150 mm in size, they are referred to as giant paraovarian cysts, a rare condition.

This article presents the case of an asymptomatic 22-year-old woman with a large pelvic mass, incidentally discovered during physical examination by her family doctor. An abdominal ultrasound was requested, and the first diagnosis was a “severely distended bladder.” The patient was referred to urology, where a neuroaxis magnetic resonance imaging (MRI) and a urodynamic study were performed, both of which turned out to be normal. Due to the inconsistency of findings, the family doctor, who was closely following the case, requested an abdominopelvic computed tomography (CT) scan, which revealed a large mass, possibly an “abdominopelvic ovarian cyst or borderline tumor.” The patient was referred to gynecology and underwent pelvic MRI. At last, a final diagnosis was made: a giant paraovarian cyst. The cyst was drained via diagnostic and curative laparotomy, followed by a quick recovery of the patient. The histopathological analysis confirmed a serous cystadenoma.

This case highlights the importance of a thorough physical examination and considering gynecologic etiologies in the differential diagnosis of pelvic masses, even in asymptomatic patients.

## Introduction

Paraovarian cysts (PCs) are cystic lesions in the broad ligament between the fallopian tube and ovary [1]. They represent approximately 10% of all adnexal masses [2-4], although their true incidence remains uncertain due to their frequent asymptomatic nature [1,4]. PCs are commonly diagnosed during the third and fourth decades of life, and up to 80% of them are diagnosed incidentally during an abdominal ultrasound [5,6].

When PCs enlarge, patients might experience pelvic pain or the feeling of a mass in the abdomen [4], but the majority of clinical signs and symptoms occur as a consequence of the pressure effect on adjacent organs or due to complications such as rupture, hemorrhage, and torsion [1,2,7]. As the mass increases in size, the risk of torsion also increases [4]. Although the overall incidence of PCs is believed to be lower in children than in adults, torsion appears to occur relatively more frequently in pediatric patients [7].

There is no universally accepted definition of giant PCs, which is a rare condition. Some authors define it as exceeding 150 mm in diameter [2,8,9], while others consider a threshold of 200 mm [2,4,10]. Traditionally, cysts are considered “large” when over 50 mm and “giant” when over 150 mm in diameter [11,12].

Diagnosis of PCs by ultrasound needs greater awareness and accuracy [13], as it is highly dependent on the operator’s experience. Consequently, inconclusive examinations or ambiguous clinical cases warrant a second opinion or further evaluation to ensure an accurate diagnosis [14].

Computed tomography (CT) or magnetic resonance imaging (MRI) proved to be more useful in differentiating PCs from ovarian cysts [4].

## Case presentation

A 22-year-old Caucasian woman with no relevant medical, family, or psychosocial history presented to the family doctor for a routine appointment. She maintained a well-balanced diet, engaged in regular physical activity, and had a body weight of 62 kg and height of 173 cm (body mass index: 20.7 kg/m^2^). She was a college student living with two roommates in a rented apartment. There was no history of previous pregnancies or abortions.

During the consultation with her family doctor, the patient denied any symptoms, stating only that she had recently modified her lifestyle to lose weight, as she had been overweight 15 months prior.

**Figure 1:**
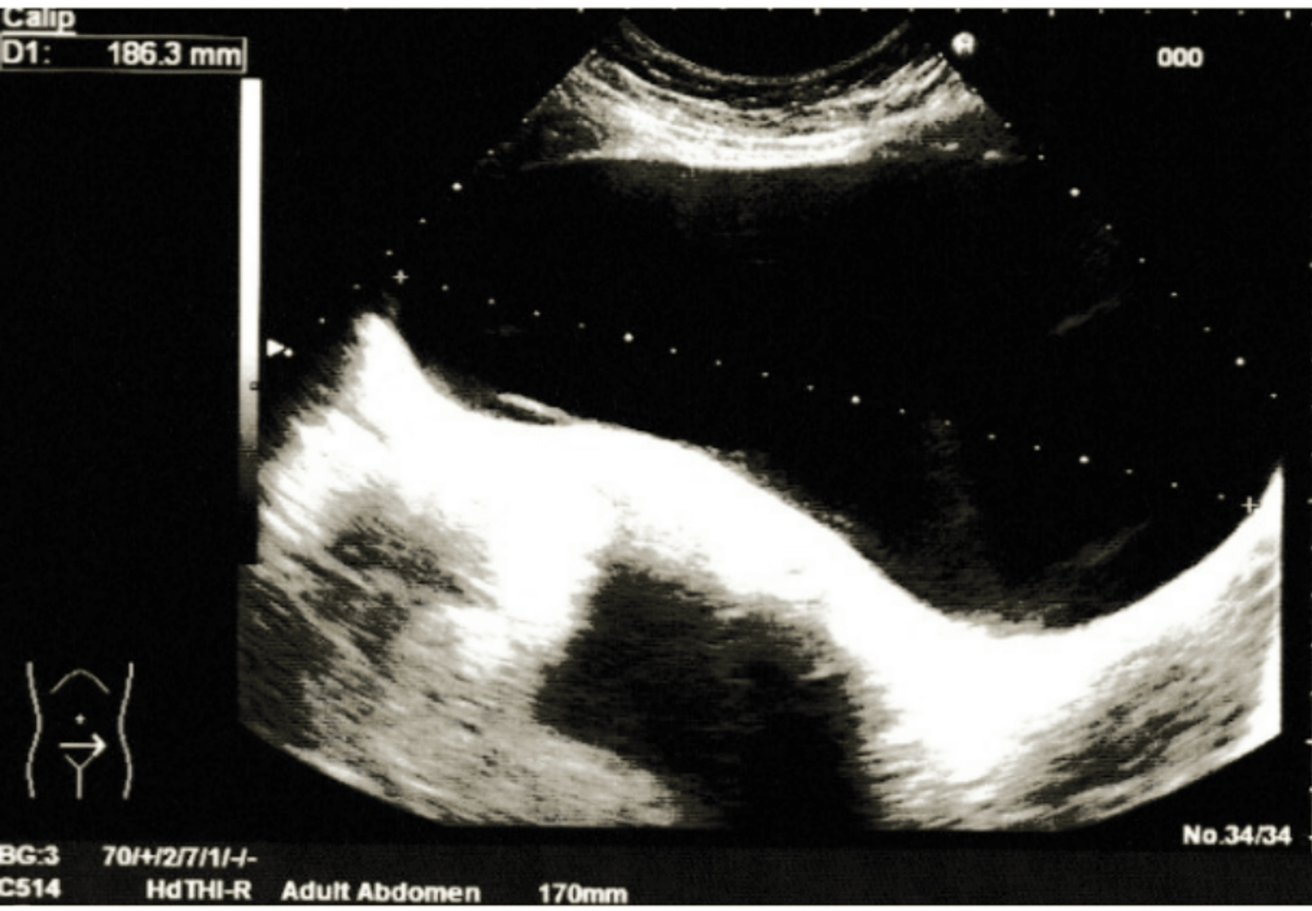
Pelvic ultrasound The image reveals a mass initially interpreted as a “distended bladder.”

**Figure 2:**
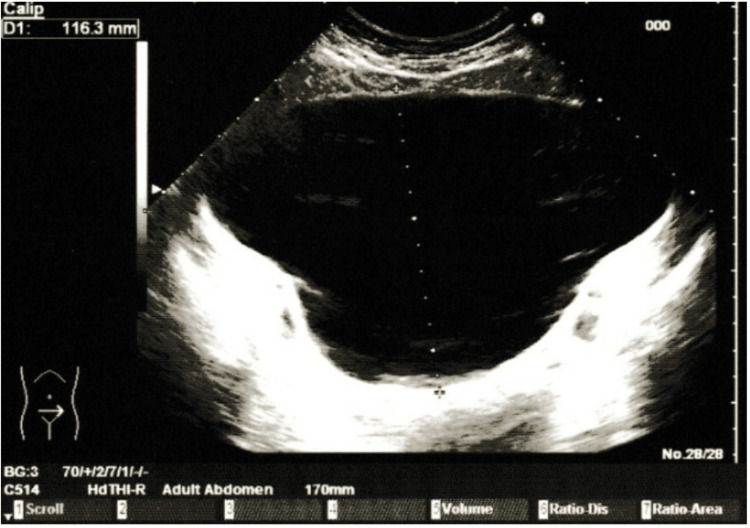
Pelvic ultrasound The image reveals a mass initially interpreted as a “distended bladder.”

A thorough and complete physical examination was performed. On abdominal examination, there was distension of the lower quadrants. Bowel sounds were present. Palpation revealed a non-mobile pelvic mass extending to the supraumbilical region. There was no tenderness or signs of peritoneal irritation. When questioned, the patient denied pregnancy, flatulence, constipation, diarrhea, or pain, and stated that it was probably residual fat. Based on these findings, a lower abdominal ultrasound was ordered and performed two weeks later. The report described: “severely distended bladder, with full size or volume unable to be determined; no wall abnormalities or content alterations noted; kidneys with normal topology and dimensions; no signs of ureterohydronephrosis” (Figure 1 and Figure 2). Consequently, the patient was referred to the urology department for further evaluation.

**Figure 3:**
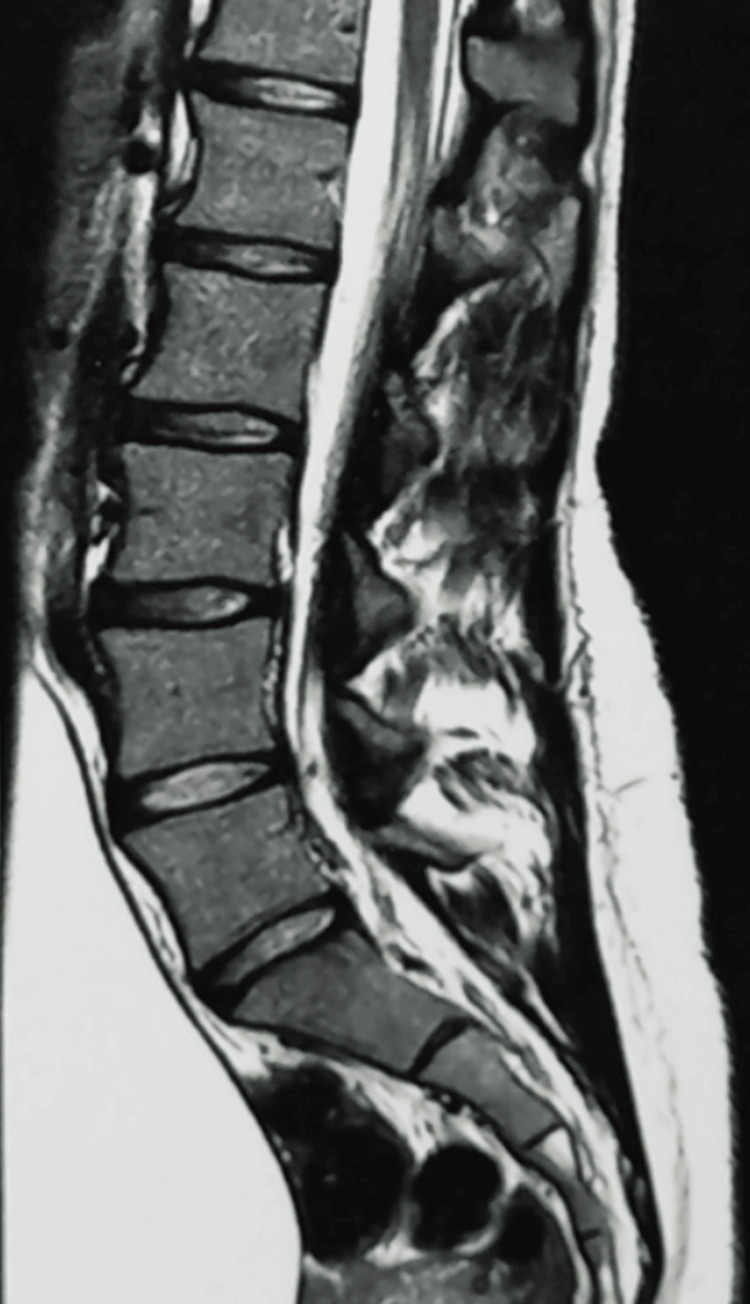
Neuroaxis MRI in sagittal section In this image of the lumbosacral region, we can observe the mass located anterior to the vertebrae. MRI: magnetic resonance imaging

Following the initial urology consultation, the possibility of a neurogenic bladder was considered. As a result, an MRI of the neuraxis and a urodynamic study were requested. In the meantime, the patient was prescribed a selective alpha-1A adrenergic receptor blocker. The MRI revealed no spinal cord lesions, tumors, or other neurological abnormalities. However, it did confirm the presence of a pelvic mass, which was also clearly visualized on this imaging modality (Figure 3).

The urodynamic study reported: “no urinary leakage with effort or coughing; negligible post-void residual volume.” In summary, neither examination identified any abnormalities that could account for the bladder distension.

In the meantime, the patient continued regular follow-up with her family doctor for general and integrated care, especially after developing anxiety related to the entire process, for which she was referred to psychology. Upon reviewing all previous examination results, the family doctor found it unusual that there was no explanation for the bladder distension and that the organ could not be fully visualized in any of the examinations. Therefore, in collaboration with the urology team, an abdominopelvic CT scan was requested in order to better characterize the mass.

**Figure 4:**
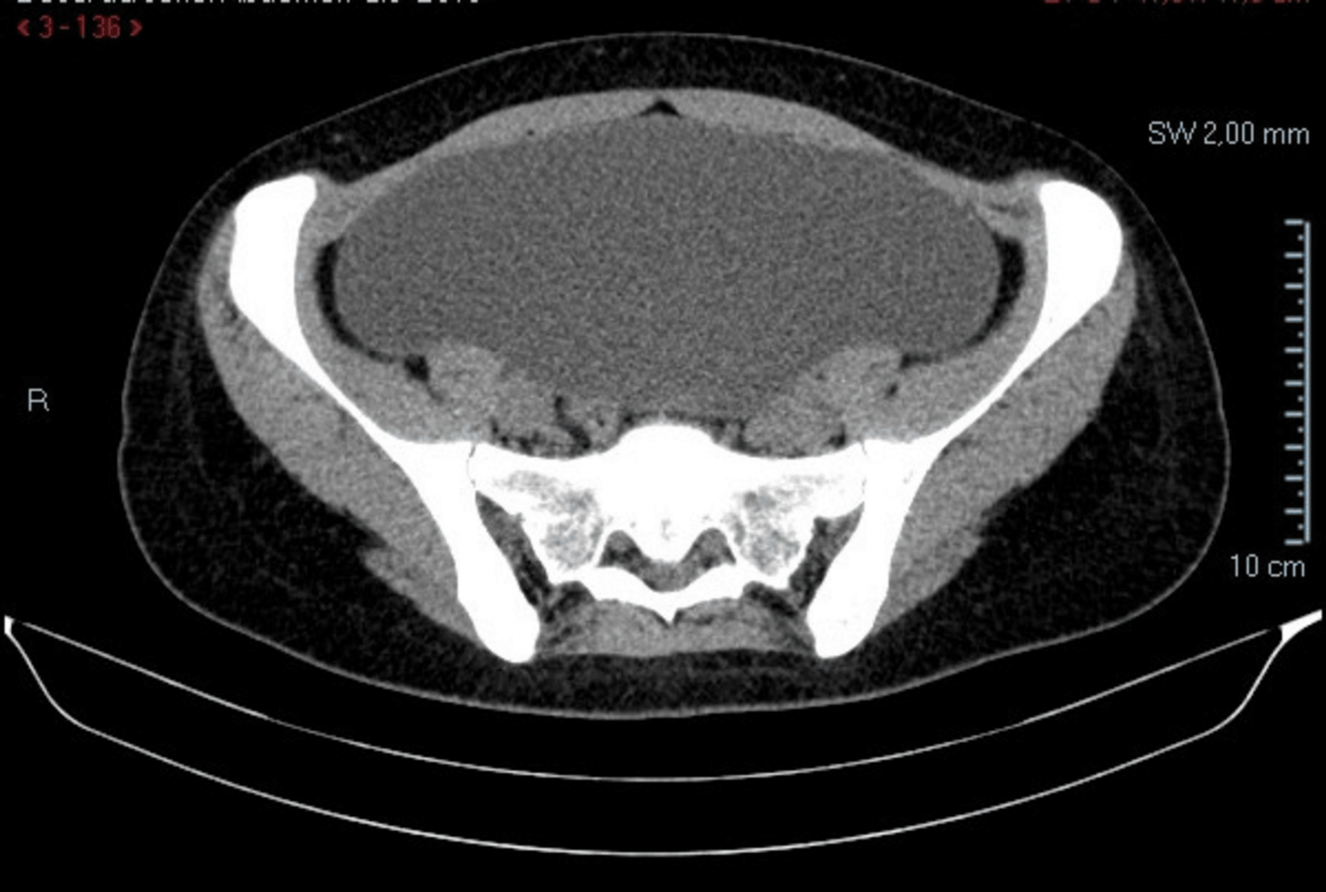
Abdominopelvic CT scan (axial section) of the cyst CT: computed tomography

**Figure 5:**
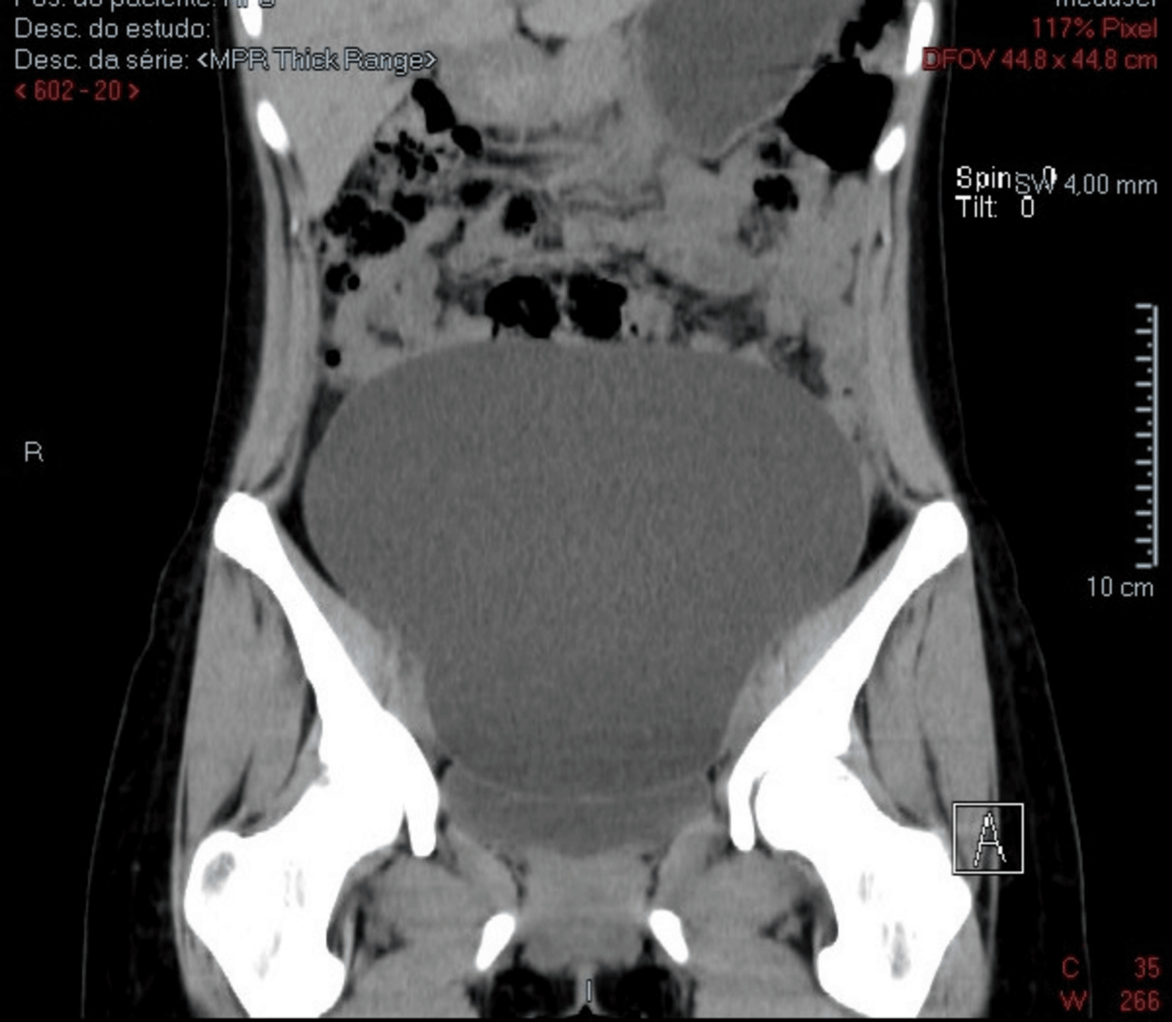
Abdominopelvic CT scan (coronal section) This image demonstrates a clear separation between the bladder and the cyst. CT: computed tomography

**Figure 6:**
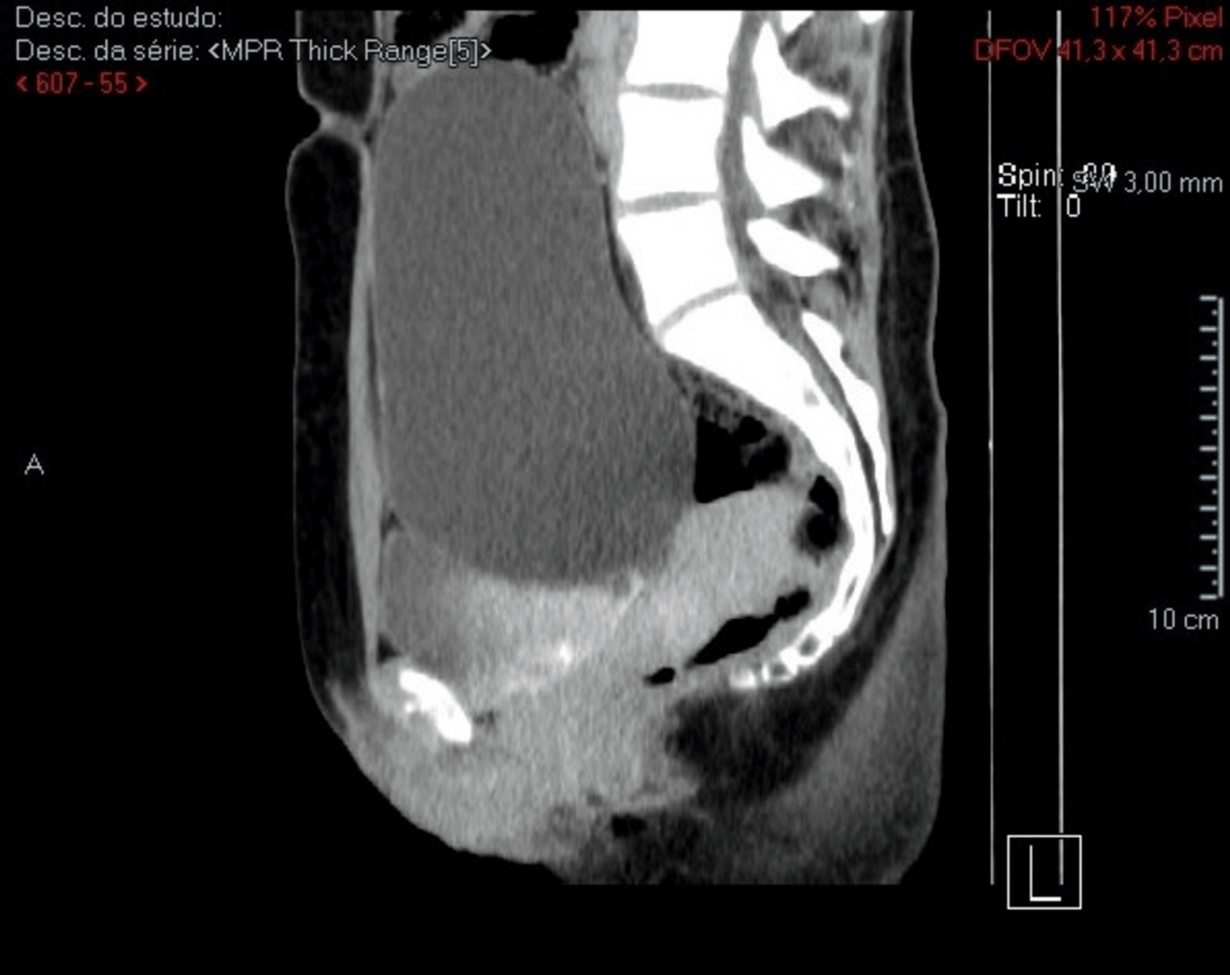
Abdominopelvic CT scan (sagittal cut) This image demonstrates a clear separation between the bladder and the cyst, especially with contrast. CT: computed tomography

The CT scan showed a “large cystic lesion centered along the midline in the abdominopelvic region, apparently originating from the left adnexa, measuring 187 × 108 × 218 mm in its longest axes (longitudinal, anteroposterior, and transverse). The wall is smoothly delineated, with no apparent septations and solid or calcified nodular components. However, given its size, histological characterization is warranted (serous cystadenoma or borderline ovarian tumor). The lesion is not related to digestive or urinary structures” (Figures 4-6).

The patient was therefore referred to the gynecology department and scheduled for surgery. Preoperative blood tests were ordered, along with an abdominopelvic MRI, which showed the following findings: “large pelvic cystic formation measuring approximately 217 × 101 × 188 mm in its greatest transverse, anteroposterior, and craniocaudal axes, respectively. The estimated volume is approximately 2150 cc. The cyst has a thin wall, without segmental thickening or irregularities, showing homogeneous intraluminal signal hyperintense on T2 and hypointense on T1, consistent with a simple cystic nature. Due to its large size, it causes significant compression of the bladder, which shows underfilling and appears to have no room for expansion beyond its current volume of approximately 25 cc. The cystic formation also causes posterior displacement of the uterus and rectum. There is no apparent direct continuity with either gonad, nor with the ileocecal appendix or the kidneys. No suspicious enhancement or diffusion restriction is observed. The uterus has a globally preserved morphology and is retroverted, due to the effect of the previously described large cystic formation. The ovaries are in their usual locations, also showing some posterior displacement. Both exhibit preserved morphostructural characteristics and dimensions within normal limits. No lymph node formations with criteria for adenopathy are identified within the chains covered by the imaging acquisition.” 

The mass was drained, and the capsule was excised via a curative laparotomy (total volume: 1700 cc). Oophorectomy was not necessary. The patient experienced a rapid postoperative recovery, and the histopathological examination revealed a serous cystadenoma.

At the postoperative gynecology consultation, the patient remained in good general condition and was asymptomatic. In subsequent appointments with her family doctor, she showed significant improvement in her anxiety symptoms and expressed relief at having reached a definitive diagnosis.

## Discussion

PCs are benign cystic lesions arising from remnants of the mesonephric or paramesonephric ducts, located in the broad ligament [1]. They account for approximately 10% of all adnexal masses and are most frequently diagnosed incidentally in women during their reproductive years, often in the third and fourth decades of life [2-6]. Most are asymptomatic and small, but when they exceed 150 mm (commonly referred to as “giant”), they become clinically significant. This classification threshold is widely accepted, although some authors recommend a higher cutoff of 200 mm [2,4,8-10]. Giant PCs may present with nonspecific symptoms or remain asymptomatic, as volume effects can compress adjacent organs, most notably the bladder, without overt urinary or gastrointestinal complaints, as seen in our case [2,5,15].

Preoperative diagnosis is a challenge due to imaging limitations: ultrasound may misinterpret large cysts as ovarian in origin or suggest bladder distension [16]. CT and MRI offer superior delineation, allowing for more accurate determination of cyst origin, size, wall characteristics, and effects on neighboring organs [4].

Complications such as torsion, hemorrhage, rupture, or borderline histology occur more frequently with large cysts [1,2,5].

Laparoscopic management with fertility preservation is increasingly advocated, particularly in young women, with studies reporting ovarian-sparing surgery in 85% and minimally invasive approaches in ~43% of cases [2].

Several case reports in adolescents and young adults corroborate the feasibility and safety of minimally invasive removal, even for massive cysts up to 400 mm, using modified laparoscopic techniques with controlled decompression to avoid spillage. However, laparotomy remains necessary if size, morphology, or suspected malignancy preclude safe laparoscopy [17-19].

As stated earlier, this case reports a healthy 22-year-old woman with no relevant medical history, who presented to her family doctor for a routine checkup. On physical examination, a non-tender pelvic mass was noted, extending to the supraumbilical region. An initial abdominal ultrasound revealed severe bladder distension with unclear limits, prompting referral to urology. MRI of the neuraxis and a urodynamic study were performed to exclude neurogenic bladder. These tests showed no neurological or urodynamic abnormalities but confirmed the presence of a pelvic mass. A CT scan revealed a large, midline cystic lesion (187 × 108 × 218 mm) suggestive of adnexal origin, with smooth walls and no septations or solid components. MRI confirmed a simple cystic structure (estimated volume: ≈2150 cc), significantly compressing the bladder and displacing the uterus and rectum. The ovaries were normally located but displaced posteriorly. No signs of malignancy or connection to adjacent organs were identified. The patient underwent diagnostic and curative laparotomy, with preservation of ovarian tissue. A total of 1700 cc of cystic fluid was drained. Histopathology confirmed a benign serous cystadenoma, consistent with the benign profile of most PCs but reinforcing the importance of surgical removal for definitive diagnosis.

This clinical case highlights the rarity and diagnostic challenge of giant PCs, which are often mistaken for other pelvic pathologies. It also emphasizes the importance of a multidisciplinary approach and advanced imaging for accurate diagnosis and appropriate management.

## Conclusions

PCs are usually diagnosed during the third and fourth decades of life, and up to 80% of them are diagnosed incidentally during a random abdominal ultrasound.

This clinical case aims to highlight the importance of a detailed clinical history and a thorough physical examination by all physicians involved in a patient’s care, even in the absence of symptoms. Furthermore, it emphasizes the value of regular follow-up with the family doctor, the importance of including gynecologic etiologies in the differential diagnosis when evaluating pelvic masses, and the need to consider the patient’s mental health in light of medical diagnoses. Equally important is the critical role of multidisciplinary collaboration across specialties in achieving accurate diagnoses and effective care. We would also like to add that ultrasound is a highly observer-dependent imaging modality and that second opinions or additional diagnostic studies may be necessary when findings are unclear or inconsistent.
